# Effects of Weight Loss on Key Obesity-Related Biomarkers Linked to the Risk of Endometrial Cancer: A Systematic Review and Meta-Analysis

**DOI:** 10.3390/cancers16122197

**Published:** 2024-06-11

**Authors:** Angela D. Clontz, Emma Gan, Stephen D. Hursting, Victoria L. Bae-Jump

**Affiliations:** 1 Department of Nutrition and Nutrition Research Institute, University of North Carolina at Chapel Hill, Chapel Hill, NC 27599, USA; adclontz@email.unc.edu (A.D.C.); hursting@email.unc.edu (S.D.H.); 2 Department of Medicine, Imperial College London, London SW7 2AZ, UK; emma.gan21@imperial.ac.uk; 3 Division of Gynecologic Oncology, University of North Carolina at Chapel Hill, Chapel Hill, NC 27599, USA

**Keywords:** endometrial cancer, cancer prevention, obesity, weight loss, inflammatory markers, hormones

## Abstract

**Simple Summary:**

Obesity significantly contributes to endometrial cancer (EC) incidence and mortality. Weight loss interventions are pivotal in mitigating endometroid EC risk, showing notable improvements in obesity-related metabolic perturbations, including insulin resistance, dyslipidemia, and inflammation. This review aimed to assess the efficacy of weight loss strategies, including lifestyle modifications, surgical interventions, and pharmacological approaches, on key biological indicators associated with endometroid EC. Findings reveal the assessment of weight loss for reducing inflammatory markers linked to endometroid EC, with bariatric surgery emerging as a prominent intervention. These insights can inform clinical practice, refine therapeutic strategies, and introduce tailored interventions to promote equitable healthcare and enhance outcomes for individuals affected by endometroid EC.

**Abstract:**

Endometrial cancer (EC) includes various histologic types, with estrogen-dependent endometrioid carcinoma being the most common. Obesity significantly increases the risk of developing this type, especially in postmenopausal women, due to elevated estrogen production by adipocytes. This review examines the impact of weight loss from different interventions on reducing obesity-related risk factors for endometrioid EC. A systematic review and meta-analysis were conducted on three weight loss interventions: bariatric surgery, pharmacotherapy, and lifestyle changes. The effects of these interventions on inflammatory biomarkers (CRP, TNF-α, IL-6) and hormones (leptin, estrogen) were analyzed. Data from controlled studies were pooled to assess the significance of weight loss in reducing these biomarkers. Despite heterogeneity, bariatric surgery resulted in an overall 25.8% weight reduction, outperforming lifestyle and pharmacotherapy interventions. Weight loss reduced CRP levels by 33.5% and IL-6 levels by 41.9%. TNF-α levels decreased by 13% with percent weight loss over 7%. Leptin levels also decreased significantly, although the exact weight loss percentage was not statistically significant. Weight loss effectively reduces proinflammatory markers and hormones associated with increased risk of endometrioid EC. The strengths of this review include a comprehensive examination of different weight-loss interventions and a large pool of participants. However, limitations include high heterogeneity among studies and only 43% of the participants being postmenopausal. Limited data on sex hormones and racial disparities underscore the need for further research.

## 1. Introduction

Endometrial cancer (EC) is a significant health concern globally, ranking as the fourth most diagnosed cancer among women [1]. Alarmingly, a profound health disparity exists in EC outcomes, with Black women experiencing a twofold increase in mortality compared to their White counterparts [2,3,4]. EC patients diagnosed with advanced-stage disease have a 5-year survival rate of ≤20% [5]. Contributing significantly to the burden of endometroid EC (or Type I EC) is obesity, which is recognized as the leading preventable risk factor for its development [6]. Obesity not only escalates the incidence and mortality rates of endometroid EC but also heightens the risk of recurrence among cancer survivors [7,8].

Understanding the intricate relationship between obesity and the risk of endometroid EC involves exploring various underlying mechanisms. For example, during menopause, the ovaries stop producing estrogen and progesterone, making adipose tissue the primary source of estrogen. Adipocytes (fat cells) convert androgens into estrogen through an enzyme called aromatase [8,9,10]. Excess adipose tissue, a characteristic of obesity, leads to increased estrogen production due to the heightened aromatization of androgens. Excess estrogen can stimulate endometrial tissue growth, contributing to the development of endometrioid EC [8]. However, other conditions, such as polycystic ovarian syndrome (PCOS) and pre- vs. postmenopausal status, which can influence ovulation and hormone levels, must also be considered when examining hormone-dependent EC risk [11].

**Figure 1:**
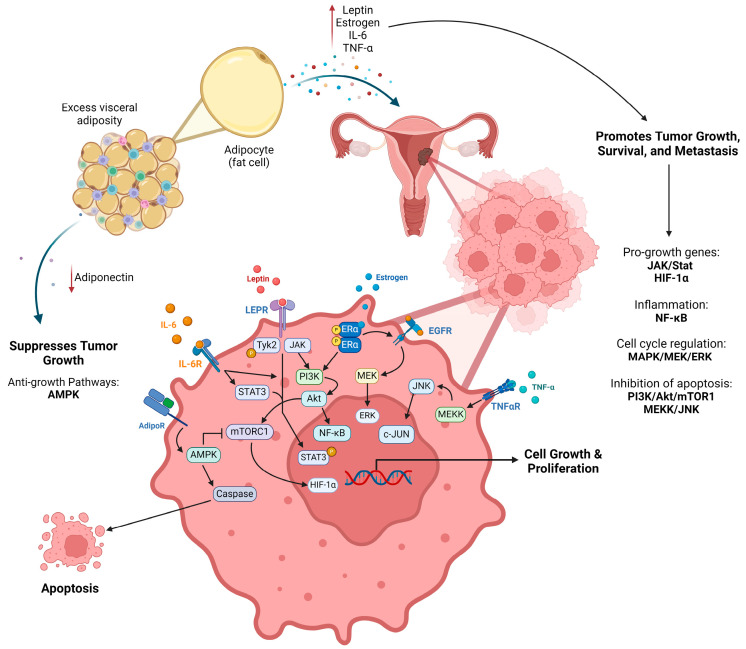
Obesity driven pathways in endometroid EC. Increased adipocytes from excess adipose tissue leads to increased levels of leptin, estrogen, IL-6, and TNF-α while reducing levels of adiponectin. Increased signaling factors (cytokines, adipokines) promote proliferation of epithelial cells of the endometrium contributing to tumorigenesis. Multiple cellular pathways, and gene expression, within epithelial cells become either overstimulated, mutated, or silenced enhancing tumor formation, survival, and metastasis. Created with BioRender.com.

The link between obesity and endometroid EC extends beyond estrogenic pathways (Figure 1). Obesity-associated changes in insulin and leptin levels contribute to dysregulated growth factor signaling and chronic inflammation, further exacerbating the estrogen-driven carcinogenic process [12,13,14,15]. Clinical studies investigating the mechanisms driving endometrioid EC have demonstrated that pro-inflammatory signaling molecules, such as leptin, IL-6, and TNF-α, are generally elevated, with tumor size and disease stage directly correlated with BMI [5,15,16,17]. Reducing pro-inflammatory signaling can ameliorate tumor-promoting pathways like PI3k/Akt/mTOR and activate tumor-suppressing pathways like AMPK in endometroid EC [18]. 

Decreasing body weight and body fat with weight loss represents a pivotal approach to reduce the risk of obesity-driven endometroid EC. Lifestyle modifications, encompassing dietary changes, increased physical activity, and behavioral interventions, serve as foundational strategies for weight management [10,19,20]. Additionally, weight loss can ameliorate obesity-related comorbidities, such as insulin resistance and chronic inflammation, further mitigating endometroid EC risk [18]. Research examining obesity and hormone-dependent breast cancer showed that an average weight loss of 5% improved obesity-related conditions, such as insulin sensitivity; however, weight loss of 10% or more significantly improved circulating levels of estradiol, adiponectin, and leptin [10,19,20].

In contrast, bariatric surgery offers a more aggressive approach for individuals with severe obesity refractory to conventional lifestyle interventions. Bariatric procedures, including gastric bypass and sleeve gastrectomy, induce excess weight loss up to 25%, often accompanied by significant improvements in metabolic parameters [20]. Emerging evidence suggests that bariatric surgery promotion of weight reduction subsequently leads to favorable changes in estrogen levels and inflammatory profiles, potentially mitigating endometroid EC risk. Studies investigating the weight-loss effect of bariatric surgery on endometroid EC showed a significant shift from a pro-inflammatory to an anti-inflammatory phenotype with weight loss of 20% or greater. Research supports the notion that weight loss achieved through bariatric surgery can lower the risk of developing endometroid EC by addressing the key physiological changes linked to obesity [9,21,22,23]. However, the optimal approach for obesity management in the context of endometroid EC remains an area of active investigation, necessitating further research to elucidate the comparative efficacy and long-term outcomes of lifestyle interventions versus bariatric surgery in this population.

Bariatric surgery is effective and provides proof of the principle that weight loss, if sufficiently significant, can reverse the pro-cancer effects of obesity. However, bariatric surgery is expensive, carries risk of adverse effects, and is only available to approximately 1% of women with obesity, so is therefore not a population-wide solution [9,24]. Life-style-based weight loss interventions are more broadly available and relatively inexpensive but are challenging for most women with obesity to sustain. Thus, emerging pharmacologic strategies that achieve and sustain significant weight loss are very promising.

Weight-loss pharmacotherapies target various physiological pathways in energy balance regulation, including appetite suppression, nutrient absorption inhibition, and metabolic modulation [25]. By augmenting satiety signals and reducing cravings, weight loss drugs facilitate adherence to calorie-restricted diets and sustain weight loss efforts. Moreover, certain weight loss medications have improved insulin sensitivity and metabolic parameters, thereby mitigating obesity-associated comorbidities, such as type 2 diabetes [26,27]. While bariatric surgery induces rapid excess weight loss and metabolic improvements, weight loss drugs offer a less invasive alternative, particularly suitable for individuals with lower BMI or those unwilling or ineligible for surgery [28,29]. However, there is a notable lack of research examining the weight-loss effects of these medications on the risk of endometroid EC, highlighting an urgent need for further investigation.

Recognizing the importance of effective weight-loss strategies to reduce body weight and visceral adiposity in the prevention of endometroid EC, this systematic review aims to evaluate the impact of weight reduction on key inflammatory biomarkers by comparing weight loss results from various interventions. Specifically, it examines whether reduced body weight can affect circulating levels of C-reactive protein (CRP), interleukin (IL)-6, tumor necrosis factor-α (TNF-α), and hormones such as leptin, adiponectin, estradiol, estrone, and testosterone. These biomarkers and hormones are known contributors to the pathogenesis of endometrioid EC. 

This review will provide valuable insights into the potential benefits of weight loss therapies as adjunctive components in the comprehensive management and prevention of EC. Thus, by delineating the landscape of interventions and their associated outcomes, this endeavor strives to inform clinical practice, enhance therapeutic strategies, and integrate tailored interventions that include multifaceted factors that are essential for advancing equitable healthcare and enhancing outcomes for all individuals affected by EC.

## 2. Materials and Methods

The protocol for this systematic review was registered with the International Prospective Register of Systematic Reviews (PROSPERO) under registration number CRD42023458858. The protocol can be accessed through the following link: https://www.crd.york.ac.uk/prospero/display_record.php?RecordID=458858 (accessed on 23 September 2023). The synthesis of reported data followed the guidelines outlined by the Preferred Reporting Items for Systematic Reviews and Meta-Analyses (PRISMA) [30].

### 2.1. Search Strategy

A thorough search strategy was executed utilizing the resources of the Health Sciences Library (HSL) at the University of North Carolina at Chapel Hill, encompassing EMBASE, PubMed, Scopus, MEDLINE, and the Cochrane Central Register of Controlled Trials (CENTRAL) databases. The search strategy incorporated a combination of keywords and Boolean operators (e.g., AND, OR) to compile comprehensive lists of relevant studies. Specific search terms employed included weight loss, endometrial cancer, bariatric surgery, lifestyle interventions, dietary intervention, and weight loss medications. The full list of search terms is presented in Supplementary Table S1.

### 2.2. Eligibility

#### 2.2.1. Study Selection

Covidence was used to manage the study selection process to screen literature that met the review criteria [31]. Records obtained from database searchers were uploaded into Covidence for title and abstract screening with duplicates automatically removed. All eligible records were fully screened, independently, by two authors (A.D.C. and E.G.). The authors resolved any screening discrepancies through discussion, or if required, consultation with the third author (V.B.-J.). 

Studies included randomized controlled trials, cohort studies, and case studies as well as retrospective and prospective studies published between 2008 and 2023. Studies must have reported pre- and post-intervention biomarkers of inflammation, adiposity, and hormones commonly elevated in patients with EC (e.g., leptin, CRP, TNF-α, IL-6, estrogen) in relation to reduced weight or BMI. Studies excluded consisted of systematic reviews, literature reviews, scoping reviews, meta-analyses, in vivo studies, in vitro studies, and publications not in English.

#### 2.2.2. Patient Selection

The inclusion criteria focused on individuals aged between 30 and 75 years, aligning with the typical age range for EC diagnosis. Furthermore, studies targeted participants with a body mass index (BMI) of 30 kg/m^2^ or higher who participated in a weight loss intervention study, encompassing lifestyle modifications, bariatric surgery, pharmacotherapy, or a combination thereof. Studies targeting participants at risk of breast cancer (BC), ovarian cancer (OC), or EC were also included. The exclusion criteria included studies involving other types of cancer, individuals with a BMI below 30 kg/m^2^, those younger than 30 years of age, or exclusively male participants. 

### 2.3. Data Extraction

The data extraction tool, designed within Covidence, captured pertinent information including author names, publication year, study country, sample size, study design, disease indication, intervention duration, and study duration. Mean baseline differences in BMI and age were recorded, along with between-group differences (intervention vs. control). Additionally, the percentage of female and Black participants was extracted. Interventions were categorized as lifestyle, bariatric surgery, pharmacotherapy, or control, with controls comprising placebo or comparison groups. 

Outcome results (e.g., weight, inflammatory markers, hormones) were tabulated pre- and post-intervention, including standard deviations (SD) and participant numbers. For consistency, mean and standard deviations were calculated for studies that only reported confidence intervals (CI) or standard error means (SEM). To ensure comprehensive coverage, additional tracking measures were instituted to limit the number of studies per outcome (e.g., maximum of 10 studies per intervention group for a single outcome). Data extraction was conducted independently by A.D.C. and E.G. following the quality assessment protocol. Any missing data were evaluated for relevance to outcomes, and authors were contacted to obtain necessary information.

### 2.4. Quality Assessment

To evaluate potential bias in randomized controlled trials, the Cochrane Risk of Bias 2.0 tool was employed, focusing on five domains: bias stemming from the randomization process, bias due to deviations from intended interventions, bias due to missing outcome data, bias in the measurement of outcomes, and bias in the selection of the reported result [32]. Nonrandomized studies, including case-control and cohort studies, were assessed using the Newcastle-Ottawa Scale (NOS) quality assessment tool [33]. Nonrandomized, or unclearly documented, studies were assessed for selection, comparability, and exposure. Studies that received ≥6 out of 9 stars were included in the analysis. Risk of bias and quality assessment analysis was conducted independently by A.D.C. and E.G. A third reviewer (V.B.-J.) was consulted to resolve any discrepancies.

### 2.5. Statistical Analysis

A comprehensive synthesis of the effect that reduced body weight has on inflammatory indicators and hormones related to the risk of EC was conducted among the different intervention groups and within the intervention groups. Averages were adjusted from the pooled data to estimate the portion of males included in the datasets to approximate female-only data (adj. avg.). 

For the meta-analysis, the random-effects model was utilized. In the random-effects model, it is presumed that the true effect differs among studies. The overall effect is determined by computing the weighted average of the observed effects from various studies. Generally, this model yields a cautious estimate, acknowledging the natural variation in the true effects found across the studies. The analysis of the extracted data for the controlled studies was performed using Cochrane Review Manager Web (RevMan Web), where outcome effect measures are reported as mean differences (MDs) with confidence intervals (CIs) [34].

### 2.6. Heterogeneity

Heterogeneity was evaluated using Cochran’s *Q* test, with a significance threshold set at less than 0.10. Additionally, the *I*^2^ statistic was employed to quantify the total observed variation across the studies. *I*^2^ value exceeding 75% was indicative of high heterogeneity, while a value below 25% suggested low heterogeneity. A value of 0% indicated no heterogeneity.

Sensitivity analysis was performed by subgrouping identified covariates within the analysis, such as the menopausal status of women, the percentage of men included in the sample, the presence of inadequate control groups, and study duration. This approach allowed for an exploration of the impact of these covariates on the overall results and provided insights into potential sources of heterogeneity.

## 3. Results

**Figure 2:**
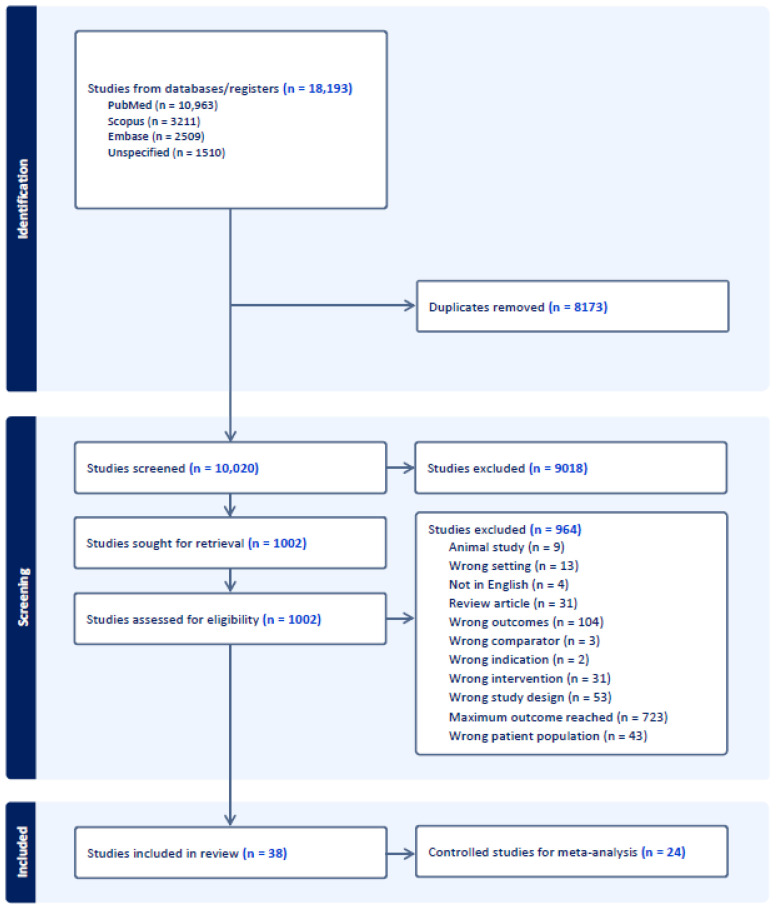
Flow diagram of the literature search.

Upon review of the online databases, a total of 18,193 abstracts were identified, with 10,963 from PubMed, 3211 from Scopus, 2509 from Embase, and 1510 from unspecified sources. Following the removal of duplicates, 10,020 unique studies remained for screening. Subsequently, upon evaluation of titles and abstracts, 9018 studies were deemed irrelevant and excluded from further consideration. This process resulted in 1002 studies being selected for full-text review to assess eligibility, leading to the exclusion of 964 studies. Thirty-eight studies remained for quality assessment and data extraction. Twenty-four studies utilizing control groups were included for the meta-analysis. A visual representation of this selection process is provided in Figure 2. 

### 3.1. Study Characteristics

The 38 unique eligible studies included (40 total studies analyzed for results of interventions due to 2 studies [35,36] incorporating both a lifestyle intervention group and bariatric surgery group as part of the study) a total of 9844 participants (male and female). The average age was 45.6 years and average BMI was 37.1 kg/m^2^. Females accounted for 81.8% (n = 8062) of the participants, with 43% (n = 3445) of the females considered postmenopausal (>50 years of age). Among the 38 eligible studies with available data on race and ethnicity, 18.9% (n = 1518) identified as Black and 76.9% (n = 6720) as White (Table 1). 

#### 3.1.1. Systematic Review

Among the 38 unique studies, investigators examined the effects of lifestyle interventions in 16 studies [12,35,36,37,38,39,40,41,42,43,44,45,46,47,48,49], bariatric surgery in 16 studies [35,36,50,51,52,53,54,55,56,57,58,59,60,61,62,63], and pharmacotherapy in 8 studies [67,68,69,70,71] (Table 1). Two studies [35,36] that evaluated both lifestyle and bariatric surgery were counted within each of those respective intervention groupings. In the lifestyle intervention group, interventions predominantly consisted of dietary modifications, exercise programs, or a combination of both, often compared to control groups. In the bariatric surgery group, fewer controls were observed, and the predominant procedure performed was gastric bypass (e.g., Roux-en-Y). In the pharmacotherapy group, medications examined for weight loss included exenatide, semgalutide, liraglutide, diacerein, beloranib, sibutramine, lorcaserin, and phentermine plus topiramate, with all studies incorporating control groups.

#### 3.1.2. Meta-Analysis

Of the 38 eligible studies, 24 unique studies were controlled (10 in the lifestyle intervention group [35,36,37,38,39,40,42,43,46,47], 9 in the bariatric surgery group [35,36,50,51,52,58,59,62,63], and 7 in the pharmacotherapy group [64,65,66,67,68,69,71]). Two studies [35,36] that evaluated both lifestyle and bariatric surgery were counted (and included in meta-analysis) within each of those respecitve intervention groupings. One controlled study with a mismatched control group (e.g., normal weight group compared to an obese weight group) was analyzed as both included and excluded from the meta-analysis [63] to demonstrate differences in the effect of the results.

For the meta-analysis, the association of weight loss on changes to inflammatory markers CRP, IL-6, TNF-α, and hormones leptin, adiponectin, estradiol, estrone, and testosterone was compared between the three different intervention strategies and/or the percent of weight loss. When heterogeneity was considered significant, subgroup analysis was performed on sex percentages (100% female (yes or no)), menopause status (pre-menopause or post-menopause), sample size (<500 or ≥500), BMI (<35 or ≥35), and intervention duration (<6 months, ≥6 months, or ≥12 months).

### 3.2. Comparison of the Effects of Different Interventions on Weight Loss

Among the 29 eligible studies reporting weight changes (11 lifestyle [12,38,39,40,41,43,44,45,46,48,49], 10 bariatric surgery [52,53,54,55,56,57,58,60,61,63], 8 pharmacotherapy [64,65,66,67,68,69,70,71]), the overall average weight loss was 13.8% (adj. avg. = 12.0%). Clear distinctions were evident among the intervention groups, with the bariatric surgery group demonstrating strong reduction in body weight, averaging 25.8% (adj. avg. = 21.6%), followed by the pharmacotherapy group with a 7.6% (adj. avg. = 6.1%) reduction and lifestyle group with an average reduction of 5.9% (adj. avg. = 4.3%).

When performing a subgroup analysis of studies that included only female participants and adjusted for menopause status, the results showed three studies (n = 1053) involving postmenopausal women and nine studies (n = 534) involving premenopausal women. The postmenopausal group consisted solely of lifestyle intervention studies, with an average weight loss of 5% [39,40,48]. The premenopausal group included two lifestyle studies, five bariatric surgery studies, and two pharmacotherapy studies [12,49,55,56,57,58,61,64,70], with an average weight loss of 22%.

#### Meta-Analysis on the Effectiveness of Weight-loss Interventions on Reducing Weight

**Figure 3:**
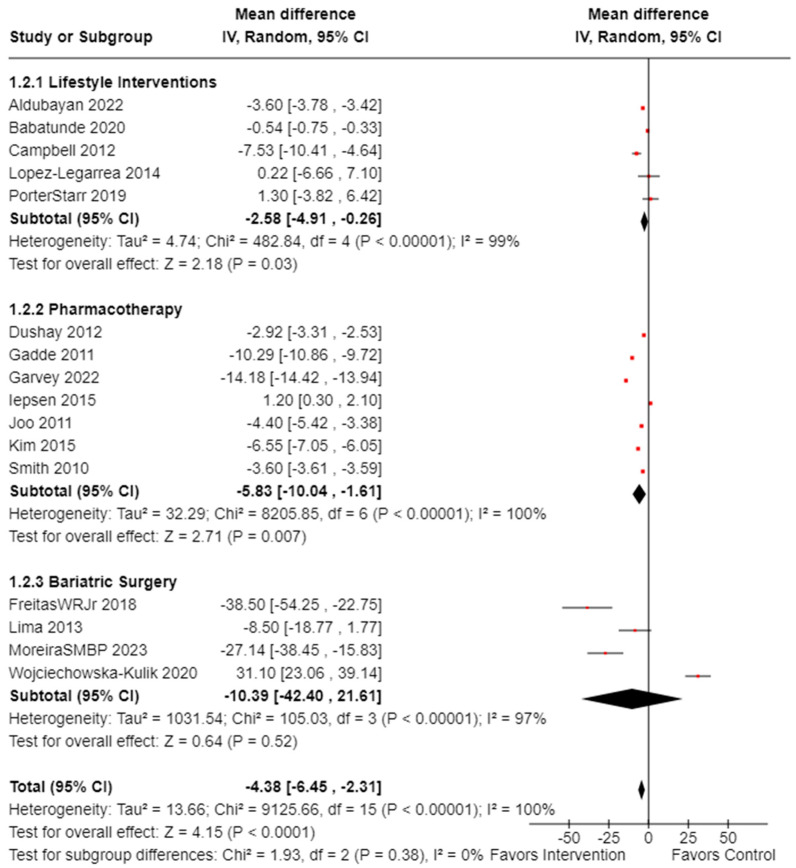
Forest plot illustrating the effects of interventions on weight loss (n = 7425). Black diamonds indicate total effect observed within groups and between groups. Red squares indicate the observed effect per individual study. Value of zero indicates no significant effect where a negative value indicates a positive effect of intervention on weight loss. Results not adjusted for female participants. **IV**: inverse variance; **CI**: confidence interval [XXX].

The first analysis evaluated the effectiveness of weight-loss interventions by examining the amount of weight participants lost. Of the 16 controlled studies reporting weight changes (n = 7425, females: 5931 or 81.5%, males: 1493 or 18.5%), the total mean difference was −4.38 (observed total effect size: 4.15; 95% CI: −6.45, −2.31; *p* < 0.0001), indicating significant effects of these interventions on weight reduction. There was significant overall heterogeneity (*I*^2^ = 100%, *p*_heterogeneity_ < 0.00001) observed, as illustrated in Figure 3 [38,39,40,43,46,52,56,58,63,64,65,66,67,68,69,71]. The analysis was repeated excluding Wojciechowska-Kulik et al. [64], who used normal weight controls. The overall effectiveness in weight loss remained statistically significant, and the weight loss effect for the bariatric surgery group demonstrated statistically significant results (observed total effect size: 2.77; 95% CI: −40.95, −6.98; *p* = 0.006).

When performing subgroup analysis of the controlled studies that included only female participants and adjusted for menopause status, the results showed two studies (n = 775) involving postmenopausal women and three studies (n = 123) involving premenopausal women. The total mean difference of weight loss for the postmenopausal group was 3.88 (observed total effect size: 1.11; 95% CI: −10.72, 2.96; *p* = 0.27) and 12.01 for the premenopausal group (observed total effect size: 1.73; 95% CI: −25.62, 1.60; *p* = 0.08), indicating menopause status was not statistically for weight loss. There was no heterogeneity between the subgroups (*I*^2^ = 8.6%, *p*_heterogeneity_ = 0.30) but high heterogeneity overall (*I*^2^ = 97%, *p*_heterogeneity_ < 0.00001) [37,39,40,42,56,58,59].

### 3.3. Comparison of Intervention-Associated Weight Loss on Inflammatory Markers

#### 3.3.1. CRP

Twenty-one eligible studies (20 unique; Abulmeaty et al. [35] counted for lifestyle and bariatric surgery) assessed changes in circulating CRP levels following weight loss interventions (7 lifestyle [12,35,38,39,43,44,46], 8 bariatric surgery [35,51,55,56,57,59,62,63], 6 pharmacotherapy [65,66,68,69,70,71]). On average, CRP decreased by 33.5% (adj. avg. = 28.7%) from baseline due to weight loss across these studies. When comparing CRP reductions across the three types of weight loss strategies, bariatric surgery resulted in the most substantial reduction in CRP levels (47%, adj. avg. = 43%), with the greatest effect demonstrated by Lima et al. [56] who reported an 89% decrease among female participants 12 months post-surgery. Weight loss from pharmacotherapy also demonstrated notable reductions (30.1%, adj. avg. = 22%), with Garvey et al. [66] reporting a 56.7% (adj. avg. = 43.9%) reduction following the administration of semaglutide for 104 weeks. In contrast, the lifestyle intervention studies showed a smaller effect of weight loss on CRP reductions (18.7%, adj. avg. = 15.7%). For example, Moszak et al. [44] showed little effect in reducing CRP levels with a hypocaloric diet with physical activity (2.4% (adj. avg. = 1.4%); however, the short duration of the intervention should be considered when assessing its effect.

##### Meta-Analysis on the Effectiveness of Weight loss on CRP

**Figure 4:**
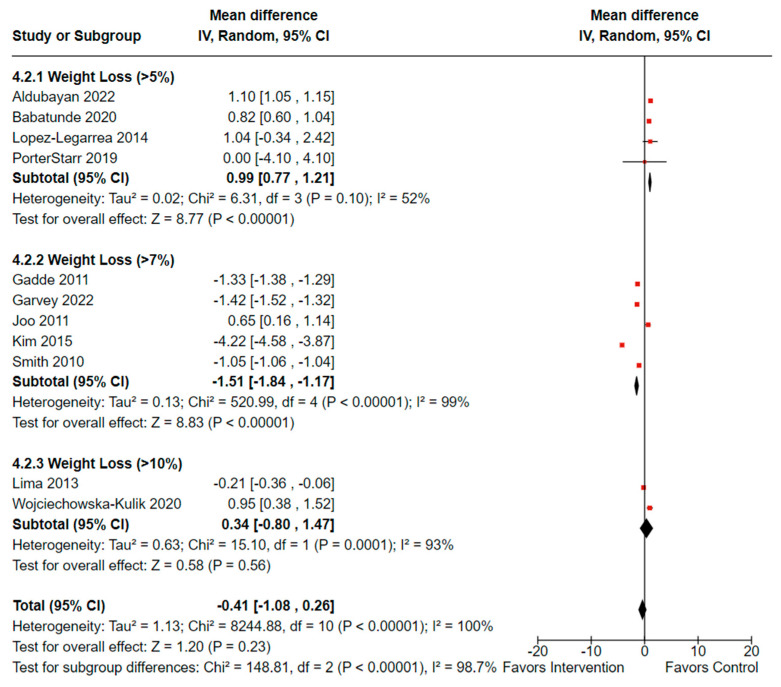
Forest plot illustrating effectiveness of weight loss on reducing CRP (n = 6762). Black diamonds indicate total effect observed within groups and between groups. Red squares indicate the observed effect per individual study. Value of zero indicates no significant effect where a negative value indicates a positive effect of weight loss on circulating CRP. Results not adjusted for female participants. **IV**: inverse variance; **CI**: confidence interval. The analysis was repeated excluding Wojciechowska-Kulik et al. [63], who used normal weight controls, and Lima et al. [56] as there was no power to assess effect size; therefore, the > 10% weight loss group was removed from the repeat analysis. The overall effectiveness of weight loss on CRP remained the same as well as the heterogeneity overall and between group differences remained high (*I^2^* = 99.3%, *p*_heterogeneity_ < 0.00001).

For the meta-analysis, changes in CRP were assessed for their association with the percentage of weight loss achieved from the interventions. Of the 11 controlled studies reporting weight changes (n = 6762, females: 5287 or 73.4%, males: 1474 or 26.5%), the total mean difference was −0.41 (observed total effect size: 1.20; 95% CI: −1.08, 0.26; *p* = 0.23). There was significant overall heterogeneity (*I*^2^ = 100%, *p*_heterogeneity_ < 0.00001) as well as significant heterogeneity between the groups (*I^2^* = 98.7%, *p*_heterogeneity_ < 0.00001), as illustrated in Figure 4. [38,39,43,46,56,63,65,66,68,69,71]. 

#### 3.3.2. TNF-α

Nine eligible studies (eight unique; Abulmeaty et al. [35] counted for lifestyle and bariatric surgery) investigated changes in circulating TNF-α levels following weight loss interventions (5 lifestyle [12,35,38,43,46], 3 bariatric surgery [35,52,62], 1 pharmacotherapy [68]), revealing an overall average decrease of 13% (adj. avg. = 5.3%) from baseline. When comparing TNF-α reductions across the three types of weight loss strategies, a bariatric surgery study conducted by Abulmeaty et al. [35] observed a substantial reduction 12 months post-gastric bypass surgery (52.3%, adj. avg. = 23.5%). In examining the lifestyle intervention studies, two out of five also showed no effect of weight loss on reducing TNF-α levels post-intervention [38,46]. Additionally, only one pharmacotherapy study investigated weight loss on TNF-α levels, reporting a 42.5% (adj. avg. = 11.9%) reduction at the end of 3 months of administering diacerein [68]. Lima et al. [56] was excluded from the analysis as sensitivity testing revealed abnormal values that were outside of the limits.

##### Meta-Analysis on the Effectiveness of Weight loss on TNF-α

**Figure 5:**
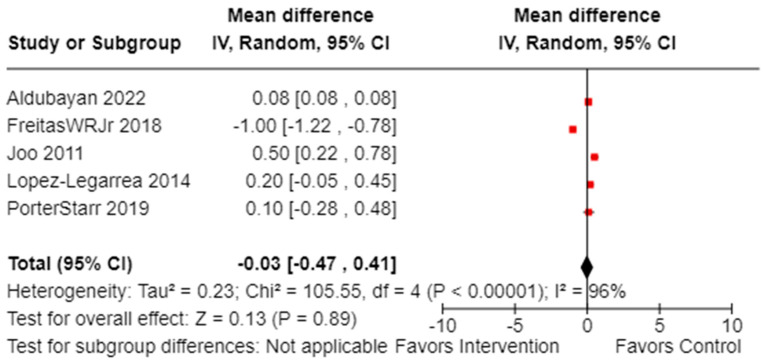
Forest plot illustrating effectiveness of weight loss on reducing TNF-α (n = 332). Black diamonds indicate total effect observed within groups and between groups. Red squares indicate the observed effect per individual study. Value of zero indicates no significant effect where a negative value indicates apositive effect of weight loss on circulating TNF-α. Results not adjusted for female participants. **IV**: inverse variance; **CI**: confidence interval.

The meta-analysis revealed that between group comparisons could not be performed as there was only one study in the >7% weight loss group and one study in the > 10% weight loss group. However, out of the five controlled studies that assessed TNF-α levels (n = 332, females: 216 or 62.2%; males: 115 or 37.8%), the total mean difference was –0.03 (observed total effect size: 0.13; 95% CI: −0.47, 0.41; *p* = 0.89), demonstrating no statistically significant effect. There was significant overall heterogeneity (*I*^2^ = 96%, *p*_heterogeneity_ < 0.00001), as illustrated in Figure 5 [38,43,46,52,68].

#### 3.3.3. IL-6

Ten eligible studies (eight unique; Abulmeaty et al. [35] and Lorenzo et al. [36] counted for lifestyle and bariatric surgery) (7 lifestyle [12,35,36,38,39,43,46], 5 bariatric surgery [35,36,51,56,57]) examined circulating IL-6 levels pre- and post-intervention, revealing weight loss caused an average reduction of 41.9% (adj. avg. = 38.2%). When comparing IL-6 reductions across the three types of weight loss strategies, four out of the five bariatric studies [35,36,51,56,57] reported that weight loss reduced IL-6 levels by an average of 46.3% (adj. avg. = 41.4%); however, MacKintosh et al. [57] reported a 95% decrease from baseline 12 months post-surgery. In the lifestyle intervention studies, the overall reported reduction of IL-6 levels was 33.5% (adj. avg. = 28.2%). The greatest effect in this group was observed by Babatunde et al. [39], who reported an 81.3% reduction in IL-6 after weight loss from a 12-month diet plus physical activity intervention, specifically in Black females with obesity. IL-6 levels were not documented in any of the eligible pharmacotherapy studies. Sensitivity analysis revealed that Tussing-Humphreys et al. [62]. had abnormal values reported and was removed from this analysis.

##### Meta-Analysis on the Effectiveness of Weight loss on IL-6

When analyzing the association of weight loss on IL-6 levels on the studies that reported weight changes, the overall findings were statistically significant. Four controlled studies (n = 922, females: 704 or 75.7%, males: 217 or 24.2%) showed a total mean difference of −0.25 (observed total effect size: 6.17; 95% CI: −0.33, −0.17; *p* < 0.00001). There was no subgroup analysis performed, as only one study with > 10% weight loss had reported data, while the >7% wight loss group had none. The heterogeneity was statistically significant among the > 5% weight loss group (*I^2^* =74%, *p*_heterogeneity_ = 0.010).

### 3.4. Comparison of Intervention-Associated Weight Loss on Hormones

#### 3.4.1. Leptin

Twenty eligible studies (6 lifestyle [37,38,41,45,46,47], 10 bariatric surgery [50,51,52,53,54,56,57,58,60,63], four pharmacotherapy [64,67,69,70]) examined pre- and post-intervention leptin levels, revealing an overall average reduction of 40% (adj. avg. = 33.2%). When comparing leptin reductions across the three types of weight loss strategies, three of ten bariatric surgery [54,56,58] studies demonstrated substantial improvements in leptin levels post-intervention exceeding 70% (adj. avg. = 64.6%). The pharmacotherapy [64,67,69,70] and lifestyle intervention [37,38,41,45,46,47] studies both reported reductions averaging 27.4% (adj. avg. = 21.4%). Notably, Lima et al. [56] and Moreira et al. [58] both reported significant leptin reductions at 78% (adj. avg. = 78%) after implementing a low-caloric liquid diet and exercise regimen for 6 weeks. Kim et al. [69], Sari et al. [70], and Iepsen et al. [67] reported 30% reductions after administering different weight loss medications.

##### Meta-Analysis on the Effectiveness of Weight loss on Leptin

**Figure 6:**
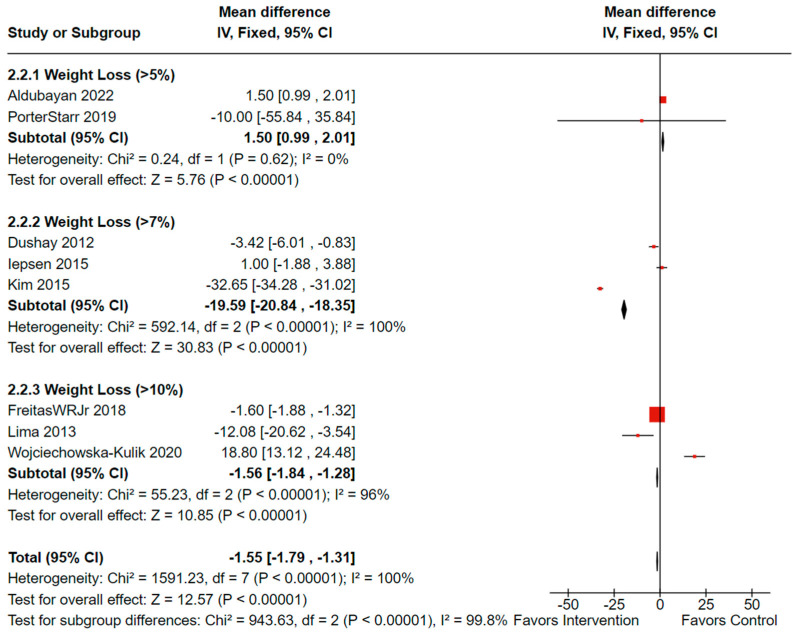
Forest plot illustrating effectiveness of weight loss on leptin levels (n = 492). Black diamonds indicate total effect observed within groups and between groups. Red squares indicate the observed effect per individual study. Value of zero indicates no significant effect where a negative value indicates a positive effect of weight loss on circulating leptin. Results not adjusted for female participants. **IV**: inverse variance; **CI**: confidence interval. The analysis was repeated excluding Wojciechowska-Kulik et al. [63], who used normal weight controls. The overall effectiveness of weight loss on leptin remained the same as well as heterogeneity for between group differences.

When analyzing the association of weight loss on leptin levels for the studies that reported weight changes, the overall findings were statistically significant. Eight controlled studies (n = 492, females: 405 or 85.3%, males: 86 or 16.5%) showed a total mean difference of −1.55 (observed total effect size: 12.57; 95% CI: −1.79, −1.31; *p* < 0.00001); however, there was significant overall heterogeneity (*I*^2^ = 100%, *p*_heterogeneity_ < 0.00001). Significant heterogeneity was also present for between group comparisons (*I*^2^ = 99.8%, *p*_heterogeneity_ < 0.00001) as illustrated in Figure 6 [38,46,52,56,63,64,67,69]. 

#### 3.4.2. Adiponectin

Fifteen eligible studies conducted pre- and post-intervention assessments of circulating adiponectin (6 lifestyle [12,37,38,41,45,46], 5 bariatric surgery [51,52,56,57,63], 4 pharmacotherapy [64,65,68,69]), the average overall increase was 22.8% (adj. avg. = 20.9%). When comparing increases in adiponectin levels across the three types of weight loss strategies, the bariatric surgery group had an increase of 54.7% (adj. avg. 51.3%) compared to an increase of 19.9% (adj. avg. 17.4%) observed in the pharmacotherapy group. The lifestyle group had mixed results. Three out of the six studies showed a decrease in adiponectin (4.5% adj. avg. 3.7%), while the other three studies showed an increase of 2.2% (adj. avg. 1.9%). Two studies were removed from the analysis after sensitivity testing revealed Kim et al. [54] and Moreira et al. [58] reported values outside of the limit.

##### Meta-Analysis on the Effectiveness of Weight loss on Adiponectin

**Figure 7:**
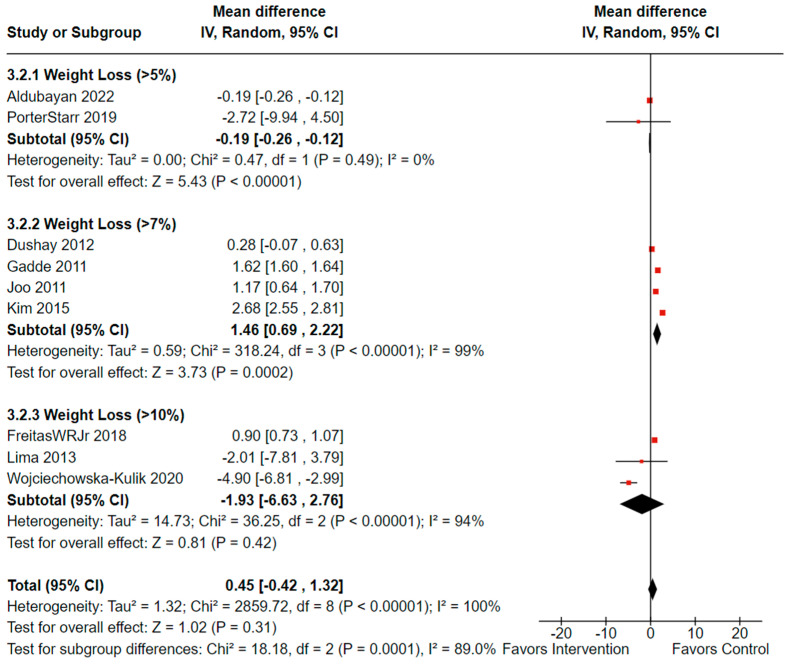
Forest plot illustrating effectiveness of weight loss on adiponectin levels (n = 2946). Black diamonds indicate total effect observed within groups and between groups. Red squares indicate the observed effect per individual study. Value of zero indicates no significant effect where a negative value indicates a positive effect of weight loss on increasing adiponectin. Results not adjusted for female participants. **IV**: inverse variance; **CI**: confidence interval. The analysis was repeated excluding Wojciechowska-Kulik et al. [63], who used normal weight controls. The overall effectiveness of weight loss on adiponectin remained the same, and heterogeneity remained significant for between group differences (*I*^2^ = 98.7%, *p*_heterogeneity_ < 0.00001).

Among the studies that reported weight loss changes and circulating adiponectin levels, nine controlled studies (n = 2946, females: 2107 or 75.7%, males: 838 or 24.2%) showed a total mean difference of 0.45 (observed total effect size: 1.02; 95% CI: −0.42, 1.32; *p* = 0.31) indicating no statistically significant effect. Furthermore, there was significant overall heterogeneity (*I^2^* = 100%, *p*_heterogeneity_ < 0.00001). Significant heterogeneity was also observed between the groups (*I^2^* =89%, *p*_heterogeneity_ = 0.0001) (Figure 7) [38,46,52,56,63,64,65,68,69]. 

#### 3.4.3. Sex Hormones

Seven eligible studies (5 lifestyle [40,42,47,48,49], 2 bariatric surgery [55,57]) reported changes in levels of sex hormones following weight loss interventions. No eligible pharmacotherapy studies assessed the weight loss effects on estradiol, estrone, or testosterone levels, precluding comment on the effectiveness of this intervention on these outcomes. After conducting sensitivity analysis, Sarwer et al. [61] was removed from the analysis for estradiol due to abnormal values that were outside the limit.

As endometroid EC is a hormone-driven cancer that is a risk factor for postmenopausal women with obesity, it is important to assess weight loss on circulating sex hormones in both pre-and postmenopausal status due to drastic hormone differences between the groups.

The subgroup analysis of six studies that included only female participants and adjusted for menopause status presented three studies (n = 1138) involving postmenopausal women [49,55,57] and three studies (n = 120) involving premenopausal women [40,42,48]. The postmenopausal group consisted solely of lifestyle intervention studies, whereas the premenopausal group was solely bariatric surgery studies. The average reduction of estradiol in postmenopausal women was 16.4%. However, estrone is the predominant form of estrogen in postmenopausal women, and the average reduction in estrone was 8.3%. Testosterone, which can be converted into estrone or estradiol, was reduced by 4.8% in postmenopausal women. More bariatric surgery studies need to assess the weight loss effects on sex hormones in this high-risk group.

##### Estradiol

Seven eligible studies (5 lifestyle [40,42,47,48,49] and 2 bariatric surgery [55,57]) reported pre-and post-intervention changes in circulating estradiol levels. When comparing changes in estradiol levels across the two weight loss groups, an average decrease was observed of 6.4% (adj. avg. = 5.8%) from baseline. A lifestyle study conducted by Shah et al. [47] reported changes in estradiol in both men and women (6.4% compared to 10.3%). One out of the two bariatric surgery studies showed a 32% decrease 12 months after surgery, while one lifestyle group showed a 21.3% decrease at 30 months post-intervention. As two studies (two lifestyle, two bariatric surgery) showed increases in estradiol, this indicates more research is needed to confirm a significant effect between the two interventions [49,57].

##### Estrone

Four eligible studies (3 lifestyle [40,42,48] and 1 bariatric surgery [55]) assessed circulating estrone levels pre- and post-intervention, showing reductions averaging 14.7%. When comparing changes across the two weight loss groups, the average reduction in estrone in the lifestyle group was 8.3%, while Kjaer et al. [55] reported a 33.8% reduction 12 months following bariatric surgery among premenopausal participants.

##### Testosterone

Seven eligible studies (4 lifestyle [40,42,48,49] and 3bariatric surgery [55,57,61]) reported pre- and post-intervention changes in circulating testosterone levels, with an average decrease of 18.1%. When comparing changes across the two weight loss groups, the bariatric surgery group showed an average reduction in testosterone from baseline of 34.3%. Sarwer et al. [61] specifically addressed the impact of bariatric surgery on sex hormones in women with obesity, showing testosterone levels decreased by 53.6% post-surgery. The two other bariatric surgery studies investigating testosterone levels showed an average decrease of 24.6% [55,57], whereas weight loss from lifestyle interventions resulted in a 5.9% decrease, and Duggan et al. [42] showed no change.

##### Meta-Analysis on the Effectiveness of Weight loss on Sex Hormones

There were only two controlled studies (lifestyle interventions only) reporting changes in sex hormones (n = 860, 100% postmenopausal women). Among the sex hormones assessed for a weight loss effect, estrone is the only hormone that demonstrated a positive effect from weight loss [40,42].

When analyzing the association of weight loss on estradiol levels for two studies that reported changes, the overall findings were not statistically significant, with a total mean difference of −3.91 (observed total effect size: 1.57; 95% CI: −8.77, 0.96; *p* = 0.12). There was significant heterogeneity among the two studies (*I^2^* =99%, *p*_heterogeneity_ < 0.00001).

When analyzing the association of weight loss on estrone levels for two studies that reported changes, the overall findings were statistically significant, with a total mean difference of −2.88 (observed total effect size: 8.07; 95% CI: −3.58, −2.18; *p* < 0.00001). There was no significant heterogeneity among the two studies (*I^2^* =0%, *p*_heterogeneity_ = 0.58).

When analyzing the association of weight loss on testosterone levels for two studies that reported changes, the overall findings were not statistically significant with total mean difference of –1.12 (observed total effect size: 0.89; 95% CI: −3.60, 1.36; *p* = 0.38). There was significant heterogeneity among the two studies (*I^2^* =89%, *p*_heterogeneity_ = 0.003).

### 3.5. Intervention Effects on Key Tumor Growth Factors

It is worth noting that one bariatric surgery study [51]), which consisted of 100% female participants, assessed pre- and post-intervention changes of VEGF. This growth factor is increased in later stages of tumor growth, including endometroid EC. Reducing levels of this protein can be an indication of a positive effect from an intervention. Dalmas et al. [51] reported a 28.1% reduction from baseline.

### 3.6. Heterogeneity

Among the 24 controlled studies evaluated for the meta-analysis, heterogeneity was pronounced in all total effects (*I*^2^ > 75%), with reduced heterogeneity observed for between group comparisons. Therefore, the random-effects model was used for all analyses. Sources for heterogeneity were analyzed using sensitivity analysis and subgroup analysis. The results of the subgroup analysis to assess sources for possible heterogeneity are available in Supplementary Table S2.

### 3.7. Bias

**Figure 8:**
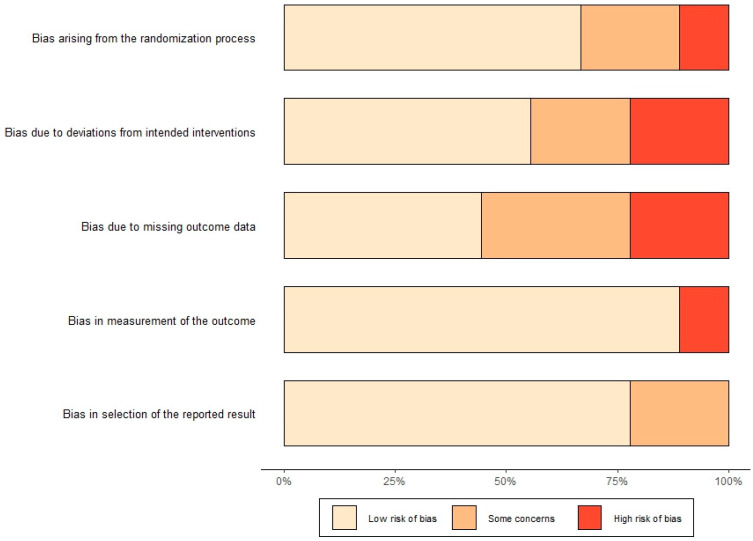
Graph displaying the RoB 2.0 five domains and risk of bias per domain for all included randomized trials (n = 25 unique studies).

There were 20 unique randomized controlled studies (Lorenzo et al. [36] was counted in lifestyle and bariatric surgery) (9 lifestyle [36,37,38,39,40,42,43,46,47], 4 bariatric surgery [36,52,56,58], 7 pharmacotherapy [64,65,66,67,68,69,71]) and 5 randomized studies (4 lifestyle [41,45,48,49], 0 bariatric surgery, 1 pharmacotherapy [70]) not utilizing controls assessed using the Cochrane Risk of Bias 2.0 (RoB 2.0) tool as shown in Figure 8. For non-randomized studies, the Newcastle-Ottawa Scale (NOS) was used to determine the quality of the studies for inclusion in the meta-analysis. The reasons for researchers not randomizing were not clarified. In the context of dietary interventions, the choice not to randomize might be due to factors such as limitations in sample size or the inherent characteristics of the intervention. Moreover, bariatric studies seldom use randomization as all participants are preselected candidates for surgery with predetermined schedules.

### 3.8. Quality

Table 2 summarizes the quality of the 14 eligible studies using the NOS scale. A study can receive up to nine stars over three categories: selection, comparability, and outcomes/exposures. For selection, four studies gained four stars, six gained three stars, and four studies gained two stars. For comparability, the controlled studies received either one or two whereas the uncontrolled studies received zero stars. For outcomes and exposures, 13 studies received three stars, one study received two stars, and zero studies received one star. Overall, the 14 studies were deemed good quality (6–9/9 stars). No study was considered fair or low quality for this analysis.

## 4. Discussion

EC is classified into various histologic types, each with distinct features and prognostic implications [72]. The most common type, endometrioid carcinoma, is linked to excess estrogen and generally has a favorable prognosis. Reducing body weight can significantly lower estrogen levels, thereby decreasing the risk and improving outcomes for estrogen-positive endometrioid carcinoma. Serous carcinoma (aggressive, not estrogen-dependent), clear cell carcinoma (rare, poor prognosis), mucinous carcinoma (rare, mucus-secreting), and carcinosarcoma (highly malignant, mixed tissue) are the other histologic types that influence prognosis and treatment strategies [72,73].

Obesity significantly increases the risk of women developing estrogen-positive endometrioid EC. This risk is particularly elevated in postmenopausal women due to increased aromatase activity in adipocytes, which converts androgens to estrogen. Excess estrogen overstimulates various pro-growth pathways, such as PI3K/Akt/mTOR, in many cells, including adipocytes and epithelial [74].

The overgrowth of cells leads to overcrowding, causing cells to become necrotic. Necrotic cells release signals, called cytokines, that alert the immune system to aid in the cleanup of cellular debris [75]. The infiltration of immune cells, particularly macrophages, leads to a state of chronic inflammation, disrupting homeostasis. This inflammatory state is characterized by the release of proinflammatory cytokines, such as TNF-α, IL-6, and CRP, which further perpetuate inflammation and, in obesity, aid in insulin resistance [76].

Inflammation from obesity is well documented as a contributing factor to several chronic, life-threatening diseases, including cardiovascular diseases, type 2 diabetes, and non-alcoholic fatty liver disease (NAFLD) [25,77,78]. In the context of cancer, chronic inflammation creates a microenvironment that promotes tumorigenesis. Elevated levels of proinflammatory cytokines lead to increased cell proliferation, angiogenesis, and inhibition of apoptosis [78]. Furthermore, the imbalance between proinflammatory adipokines, like leptin, and anti-inflammatory adipokines, like adiponectin, exacerbates metabolic disturbances, increasing the risk of developing endometroid EC [79]. Addressing obesity and its related inflammation through weight loss can be crucial in reducing the incidence of endometroid EC.

This systematic review and meta-analysis examined the impact of weight loss strategies on reducing body weight, which can reduce body fat, waist circumference, and BMI, to reduce the risk of developing endometrioid EC. Reducing these anthropometric outcomes can help reduce the incidence of inflammatory biomarkers that can promote endometroid EC development. Reducing this risk is particularly important for postmenopausal women, who are at greater risk of developing obesity, heightening their risk in developing endometroid EC [17]. However, weight loss is not the only determinant in reducing endometroid EC. Decreases in endometrial stimulation by estrogen also need to be assessed.

For this assessment, three weight loss strategies—bariatric surgery, pharmacotherapy, and lifestyle interventions—were analyzed to determine whether weight loss reduced circulating levels of CRP, TNF-α, IL-6, leptin, and estrogens, all of which are considered markers of increased endometrial proliferation and inflammation due to obesity. The results suggest that bariatric surgery has a 25.8% reduction in weight higher than lifestyle or pharmacotherapy interventions. The meta-analysis suggests that any intervention is significant at reducing body weight (*p* < 0.0001). It is important to note that bariatric surgery may not be an option for everyone. Surgery requires a lengthy recovery time and an increased risk for other health complications, and it can take from months to years to achieve the long-term benefits. For this reason, other alternatives need to be considered to prevent hormone-dependent endometrial hyperplasia, such as nutritional, behavioral, and estrogen-progesterone therapies.

The most notable biomarkers of inflammation linked to obesity and endometroid EC—CRP, IL-6, TNF-α, and leptin—were assessed to determine whether weight loss reduces their circulating levels across the three interventions. Overall, the results suggest that weight loss reduces CRP levels by 33.5%, with weight loss >7% having the most significant effect (*p* < 0.00001). The results also suggest that weight loss also improves IL-6 levels, with an average reduction of 41.9%. These results were confirmed by the meta-analysis showing a significant on IL-6 levels effect due to weight loss (*p* < 0.00001). Regarding TNF-α, the meta-analysis showed no significant overall effect; however, weight reductions of 10% or greater did suggest a significant effect (*p* < 0.00001) confirming the results of the systematic review showed an average decrease of 13%.

Leptin, an adipokine secreted by adipocytes, is significantly involved in the progression of estrogen-dependent cancers, such as EC. Elevated leptin levels, common in obesity, are linked to several oncogenic mechanisms. Leptin promotes angiogenesis by stimulating the production of vascular endothelial growth factor (VEGF), which supports tumor growth by enhancing blood supply [80]. It also activates pro-growth signaling pathways like JAK/STAT and PI3K/AKT, leading to increased cancer cell proliferation and migration [81]. Furthermore, leptin fosters a proinflammatory tumor microenvironment by increasing cytokine secretion and immune cell infiltration, which can enhance estrogen receptor activity and tumor progression [82]. Additionally, leptin inhibits apoptosis by inhibiting the AMPK pathway, allowing for uncontrolled cell growth [80].

Weight loss is an opportunistic approach to improve the ratio of circulating leptin and adipokine levels, thereby reducing factors that promote metabolic disorders and increase the risk of EC. Results from the meta-analysis suggest that weight loss has a significant effect on reducing leptin levels *(p* < 0.00001) with any percentage of weight loss improving circulating levels. The same suggestion holds true for weight loss up to 7% improving levels of anti-inflammatory adiponectin; however, weight loss over 10% did not have as significant of an effect (*p* = 0.42) suggesting weight loss overall may not improve circulating adiponectin (*p* = 0.31).

Cytokines and adipokines are not the only indicators of inflammation and imbalanced homeostasis related to obesity and increasing the risk of endometroid EC. Various sex hormones also play roles in pro- and anti-inflammatory processes that obesity can exacerbate, leading to cancer development, such as estrogen.

In postmenopausal women, high levels of estrogen are a profound contributor to the increased risk of obesity contributing to endometroid EC. Estrogen drives many cell proliferation pathways, including PKA/CREB and PI3K/Akt/mTOR that are known to be upregulated in EC [81]. Weight loss can directly reduce estrogen levels by reducing body fat percentages and visceral adiposity, as demonstrated by Campbell et al. [40] and Duggan et al. [42]. Estrone, a weaker form of estrogen, is more prominent in postmenopausal women, who are at higher risk for EC. Therefore, reducing visceral adiposity can affect circulating estrone levels [83].

In the meta-analysis, only two studies assessed the impact of weight loss on circulating sex hormones, all within the lifestyle intervention group. This highlights the need for more controlled studies measuring sex hormones as outcomes in obesity and weight loss research. Overall, the reduction in estradiol from weight loss was not statistically significant (*p* = 0.12). Estrone was significantly reduced by weight loss in both studies that examined this outcome (*p* < 0.00001). And testosterone, which can be converted into estradiol or estrone by aromatase, had similar findings to estradiol: no overall effect of weight loss (*p* = 0.38) [40,42]. Due to the limited number of controlled studies and varied heterogeneity, a definitive conclusion on the effectiveness of weight loss on reducing sex hormones to decrease the risk of EC remains to be elucidated.

Overall, the conclusions from this review and meta-analysis suggest that weight loss interventions, in general, promote significant weight reductions that can reduce proinflammatory markers and hormones that are implicated in the risks of developing endometroid EC. However, the outcomes from this review must be interpreted with caution due to the large amount of heterogeneity observed among the included studies. For example, the pharmacotherapy group sparsely reported, or did not report, any outcomes related to TNF-α, IL-6, leptin, adiponectin, and sex hormones affecting between group comparisons and overall effects. In addition, the high heterogeneity among the ages and sex of the participants was a limiting factor in correlating the weight-loss effect on the specific risk to postmenopausal women. As premenopausal women are protected by progesterone produced by the ovaries, some of the effects observed in women may be related to intact ovaries as well as other lifestyle conditions and underlying diseases.

An important observation worth addressing in this review is the underrepresentation of Black participants across the weight loss studies examined, as demonstrated by the fact that only 18.9% of the participants identified were Black, while White participants represented 76.9%. Out of the 38 unique studies, only 14 included Black participants (7 lifestyle [37,39,40,42,45,46,48], 5 bariatric surgery [52,58,59,61,62], 3 pharmacotherapy [65,66,71]), while the other studies did not disclose race or only enrolled a specific race that excluded Black participants. Studies conducted in the US, the UK, and Brazil mostly reported race, while studies in other countries typically did not.

The lack of diversity in study samples not only hampers the generalizability of research findings but also perpetuates health disparities, particularly in conditions such as obesity and EC, where Black individuals experience disproportionate burdens [84]. The understanding of weight loss interventions to reduce the risk of cancer or other co-morbidities is critical, especially with the high rate of mortality from endometrioid EC observed in Black women [2,4]. Only one eligible study included in this review, Babatunde et al. [40], consisted of 100% Black, female participants with obesity (n = 336) to address the lack of research available in this population. Interestingly, in this randomized controlled study, a 12-month diet and exercise intervention had little to no effect on weight loss (0.3%) but did reduce proinflammatory markers IL-6 (81.3%) and CRP (15.7%) [39]. This data warrant that weight loss is not a “one size fits all” solution, and culture, race, and ethnicity may influence response. Not addressing race as a factor in research undermines efforts to develop effective interventions and treatments tailored to the needs of diverse populations and hinders the identification of culturally appropriate strategies for weight management and prevention of obesity-related diseases among Black individuals. 

The strengths of this study include a comprehensive examination of the effects of weight loss from different types of interventions on key biomarkers related to obesity and the risk of endometroid EC. Another strength is the large pool of participants and the high proportion of included females with the comparison of weight loss on sex hormones. This review also identifies significant gaps in current research, such as the underrepresentation of cytokine, adipokine, and sex hormone outcomes in many of the weight loss interventions but especially in pharmacotherapy. Additionally, it highlights the lack of diversity in study sampling, which impacts the generalizability of weight loss strategies, particularly in addressing health disparities among participants with obesity who are at greater risk of developing endometroid EC.

There are also several limitations of this study that have been identified. The limited number of controlled studies, especially in the context of sex hormones and pharmacotherapy, restricts the ability to draw definitive conclusions. Additionally, there is variable efficacy among different interventions, with lifestyle modifications showing limited effectiveness in some cases, suggesting the need for more robust and sustained strategies. Furthermore, the included studies varied in sample size, intervention duration, and follow-up periods, which can impact the overall conclusions and consistency of the findings.

## 5. Conclusions

This review highlights the intricate relationship between obesity and EC pathogenesis, emphasizing the need for comprehensive and inclusive approaches to improve outcomes across diverse populations. Future pharmacotherapy research is crucial to investigate the effects of weight loss on common proinflammatory indicators and hormones linked to obesity and EC, given their pivotal roles in cancer-related pro-growth pathways. The lack of assessment of weight loss effects on these indicators emphasizes a significant gap in current knowledge.

Various weight loss strategies, including bariatric surgery, lifestyle interventions, and pharmacotherapy, have been explored to evaluate their impact on EC risk. Bariatric surgery, known for its efficacy in weight reduction, is promising in lowering the risk of endometroid EC due to decreases in body weight that can lead to reductions in fat mass and BMI, which are favorable hormonal alterations. However, most women with obesity do not have access to bariatric surgery due to cost, adverse effects, or exclusion criteria. In contrast, lifestyle interventions, which promote gradual weight loss, and less invasive approaches have had smaller reductions in the percent of weight lost (5% or less) that indicate more innovative solutions are warranted.

Pharmacological approaches with weight loss drugs represent a less invasive alternative to bariatric surgery, with increased weight loss over lifestyle interventions, but they are expensive, have unwanted gastrointestinal side effects, and currently, there is little research on their effectiveness in reducing endometroid EC. This represents a critical need to understand the potential of pharmacologic weight-loss interventions more fully, such as incretin mimetics, for EC risk reduction.

This study significantly contributes to understanding the relationship between obesity, weight loss, and the risk of endometroid EC. However, several limitations warrant consideration. The high heterogeneity among included studies, influenced by variations in sample sizes, intervention durations, and follow-up time points, presents a challenge to interpretation. The limited number of studies reporting on inflammatory and hormone outcomes, particularly in the pharmacotherapy group, may impact overall conclusions. Additionally, the inclusion of both males and females necessitates estimating averages for females, potentially affecting the precision of results. Nonetheless, a notable strength of the study is the extensive participant pool, with a high proportion of females, allowing for relevant comparisons within a high-risk population for risk of endometrioid EC. Moreover, the study is groundbreaking in its comprehensive analysis of major weight loss interventions alongside the latest pharmacotherapies, providing valuable insights into their relative efficacy.

Future research should focus on standardizing intervention methods and controlling for confounding variables to better understand the relationship between weight loss and circulating inflammatory biomarker levels. This review provides valuable insights into the potential advantages of weight loss therapies as supplementary elements in the holistic treatment of EC. By outlining interventions and their outcomes, this endeavor seeks to enhance clinical practices, optimize therapeutic approaches, and incorporate customized interventions that address various factors essential for promoting equitable healthcare and improving outcomes for individuals impacted by EC.

## Figures and Tables

**Table 1:** Characteristics of included studies that examined weight loss strategies for obesity.

Study Name (Year) Country	Study Design	Intervention	Population (Intervention/Control)	Biomarkers Measured Pre- and Post-Intervention	BMI (Mean)	% Female	% Black	% White	Age (Mean)	Intervention Length
**Intervention: Lifestyle**										
* Abbenhardt (2013) USA [37]	RCT	Diet + exercise Exercise Diet Control	Women aged 50 to 75 (n = 116/117/118/87)	BMI; leptin; adiponectin	30.9	100	8	92	57.9	12 months
* Abulmeaty (2023) Saudi Arabia [35]	CT	Diet + exercise Control	Adults aged 18 to 60 (n = 14/24)	BMI; CRP; IL-6; TNF-α	40.9	45	NR	87	35	6 months
* Aldubayan (2022) Denmark [38]	RCT	Diet + exercise Control	Adults (n = 49/51)	BMI; weight; leptin; CRP; IL-6; adiponectin; TNF-α	32.2	69	NR	NR	45.3	2.5 months
* Babatunde (2020) USA [39]	RCT	Diet + PA Control	Adult women (n = 176/161)	BMI; weight; CRP; IL-6	39.1	100	100	0	49.5	12 months
* Campbell (2012) USA [40]	RCT	Diet + exercise Exercise Diet Control	Women aged 50 to 75 (n = 117/117/118/87)	BMI; weight; estradiol; estrone; testosterone	30.9	100	8	92	57.9	12 months
Claessens (2009) Netherlands [41]	RT	CR 6 weeks: HC maintenance HPC maintenance HPW maintenance	Adults aged 30 to 60 (n = 20/20/20)	BMI; weight; leptin; adiponectin	32.9	52	NR	NR	45.4	4.5 months
* Duggan (2019) USA [42]	RCT	Diet + exercise Control	Women aged 50 to 75 (n = 151/270)	BMI; estradiol; estrone; testosterone	30	100	13	85	58.5	30 months
Gomez-Huelgas (2019) Spain [12]	Cohort	Diet + Exercise	Adult women (n = 115)	BMI; weight; CRP; IL-6; adiponectin; resistin; TNF-α	36.3	100	NR	NR	44.5	24 months
* Lopez-Legarrea (2014) Spain [43]	RCT	RESMENA Diet Control	Adults (n = 48/48)	BMI; weight; TNF-α; IL-6; CRP	35.9	50	NR	NR	50	2 months
* Lorenzo (2022) Spain [36]	RCT	VLCKD RESMENA Diet Control	Adults (n = 20/20/32)	BMI; IL-6	35.7	63	NR	NR	40.2	6 months
Moszak (2018) Poland [44]	Cohort	PA + hypocaloric diet	Adults (n = 24)	BMI; weight; CRP	39.7	58	NR	NR	46	<1 month
Pinto (2020) UK [45]	RT	CER IER	Adults aged 35 to 75 (n = 22/21)	BMI; weight.; leptin; adiponectin	31	72	10	90	53	1 month
* Porter Starr (2019) USA [46]	HCT	HP TWL (control)	Adults aged 65 or older (n = 25/14)	BMI; weight; adiponectin; leptin; IL-6; CRP; TNF-α	37	31	11	73	68.3	6 months
* Shah (2011) USA [47]	RCT	Diet + exercise Exercise Diet Control	Adults aged 65 or older (n = 28/26/26/27)	BMI; leptin; estradiol	37.8	63	NR	NR	69.8	12 months
Stolzenberg-Solomon (2012) USA [48]	RT	PC IT	Women aged 50 or older (n = 105/173)	BMI; weight; estradiol; estrone; testosterone	33.4	100	37	62	59.3	18 months
Swora-Cwynar (2016) Poland [49]	RT	LC IM	Women aged 18 to 40 (n = 39/38)	BMI; weight; estradiol; testosterone	38.1	100	NR	NR	31.4	3 months
**Study name (year) country**	**Study design**	**Intervention details**	**Population (intervention/control)**	**Biomarkers measured** **Pre-and post-intervention**	**BMI (mean)**	**% Female**	**% Black**	**% White**	**Age (mean)**	**Intervention length**
**Intervention: Bariatric Surgery**										
* Abulmeaty (2023) Saudi Arabia [35]	CT	SG Control	Adults aged 18 to 60 (n = 18/24)	BMI; CRP; IL-6; TNF-α	40.9	45	NR	NR	35	6 months
Ceccarini (2019) Italy [50]	CT	GB Lean control Surgical control	Adults aged 24 to 59 (n = 51/41/9)	BMI; leptin	48.1	64	NR	NR	47.7	18 months
* Dalmas (2011) France [51]	CT	RYGB Control	Adult women (n = 51/14)	BMI; leptin; adiponectin; TNF-α; IL-6; CRP; VEGF	36	100	NR	NR	41.2	24 months
* Freitas (2018) Brazil [52]	RCT	GB Control	Adults aged 18 to 65 (n = 55/14)	BMI; weight; leptin; adiponectin; TNF-α	47.1	85	22	78	41.3	6 months
Jacobsen (2012) Denmark [53]	Cohort	GB	Adults aged 20 to 60 (n = 8)	BMI; weight; leptin	46.7	75	NR	NR	35.5	<1 month
Kim (2023) Korea [54]	Cohort	GB	Adults aged 20 to 65 (n = 63)	BMI; weight; leptin; adiponectin; Resistin	38.9	70	NR	NR	37.5	12 months
Kjaer (2017) Denmark [55]	Cohort	RYGB	Women aged less than 50 (n = 31)	BMI; weight; CRP; estradiol; estrone; testosterone	44.1	100	NR	NR	34	12 months
* Lima (2013) Brazil [56]	RCT	RYGB Control	Women aged less than 50 (n = 10/10)	BMI; weight; leptin; adiponectin; resistin; IL-6; TNF-α; CRP	45.65	100	NR	NR	35.9	15 months
* Lorenzo (2022) Spain [36]	RCT	Laparoscopic Control	Adults (n = 39/32)	BMI; IL-6	45.6	63	NR	NR	40.2	6 months
MacKintosh (2019) UK [57]	Cohort	GB SG	Adult women (n = 72)	BMI; weight; leptin; CRP; IL-6; adiponectin; estradiol; progesterone; testosterone	52.1	100	NR	NR	42	12 months
* Moreira (2023) Brazil [58]	RCT	GB Control	Women aged 18 to 65 (n = 64/11)	BMI; weight; leptin; adiponectin;	47	100	12	88	42.3	6 months
* Moriconi (2022) Italy [59]	CT	RYGB Control	Adults aged 18 to 65 (n = 50/11)	BMI; leptin; adiponectin	47	100	12	NR	42.3	6 months
Nikolic (2011) Croatia [60]	Cohort	IGB	Adults aged 20 to 60 (n = 43)	BMI; weight; leptin	41.1	82	0	NR	35	12 months
Sarwer (2018) USA [61]	Cohort	GB	Adult women (n = 106)	BMI; weight; estradiol; testosterone	44.5	100	3	97	41	48 months
* Tussing-Humphreys (2011) USA [62]	CT	GB Control	Adult women (n = 20/20)	BMI; CRP; IL-6, TNF-α	46.6	100	48	52	36.3	6 months
* Wojciechowska-Kulik (2020) Poland [63]	CT	IGB Control	Adults (n = 30/18)	BMI; weight; leptin; adiponectin; CRP	40.9	57	NR	NR	41.2	6 months
**Study name (year) country**	**Study design**	**Intervention details**	**Population (intervention/control)**	**Biomarkers measured** **Pre-and post-intervention**	**BMI (mean)**	**% Female**	**% Black**	**% White**	**Age (mean)**	**Intervention length**
**Intervention: Pharmacotherapy**										
* Dushay (2012) USA [64]	RCT	Exenatide Placebo	Women aged 18 to 70 (n = 21/21)	BMI: weight; leptin; adiponectin	33.1	100	NR	NR	48	9 months
* Gadde (2011) USA [65]	RCT	Phen/top 7.5/46 mg Phen/top 15/92 mg Placebo	Adults aged 18 to 70 (n = 498/995/994)	BMI; weight; CRP; adiponectin	36.5	70	11	86	51.1	14 months
* Garvey (2022) USA [66]	RCT	Semaglutide Placebo	Adults aged 18 or older (n = 152/152)	BMI; weight; CRP	38.6	78	4	96	47.4	26 months
* Iepsen (2015) UK [67]	RCT	Liraglutide Placebo	Adults aged 18 to 65 (n = 27/25)	BMI; weight; leptin	30.8	85	NR	NR	46	12 months
* Joo (2011) Korea [68]	RCT	Diacerein Placebo	Adults aged 20 or older (n = 12/7)	BMI; weight; CRP; adiponectin; TNF-α	31	28	NR	NR	38	3 months
* Kim (2015) Australia [69]	RCT	Beloranib Placebo	Adults aged 18 to 65 (n = 109/38)	BMI; weight; leptin; CRP; adiponectin;	37.6	93	NR	NR	48.3	3 months
Sari (2010) Turkey [70]	RT	Sibutramine Sibutramine + metformin	Adults aged 18 to 65 (n = 36/34)	BMI; weight; leptin; CRP	39.8	100	NR	NR	46.9	12 months
* Smith (2010) USA [71]	RCT	Lorcaserin Placebo	Adults aged 18 to 75 (n = 1595/1587)	BMI; weight; CRP	36.2	83.5	19	81	44.1	12 months

**CER:** continuous energy restriction; **CR**: calorie restriction; **CT**: controlled trial; **GB**: gastric bypass; **HC**: high carbohydrates; **HCT**: historically controlled trial; **HP**: high protein; **HPC**: high-protein casein; **HPW**: high-protein whey; **IER**: intermittent energy restriction; **IGB:** intragastric balloon; **IM**: isocaloric + metformin; **IT**: interactive technology; **LC**: low calorie; **NR**: not reported; **PA**: physical activity; **PC**: personal contact; **Phen/top**: phentermine plus topiramate; **RCT**: randomized controlled trial; **RT**: randomized trial; **SG**: sleeve gastroplasty; **RYGB**: Roux-en-y gastric bypass; **TWL**: traditional weight loss; **VEGF**: vascular endothelial growth factor; **VLCKD**: very low-calorie ketogenic diet. * Included in meta-analysis.

**Table 2:** Quality assessment using the Newscastle-Ottawa Scale (NOS). “*” is an awarded star.

Study	Selection	Comparability	Outcomes/Exposure	Total
Abulmeaty, 2023 [35]	****	*	***	8
Ceccarini, 2019 [50]	***	*	**	6
Dalmas, 2011 [51]	**	*	***	6
Gomez-Huelgas, 2019 [12]	***		***	6
Jacobsen, 2012 [53]	****		***	7
Kim, 2023 [54]	****		***	7
Kjaer, 2017 [55]	***		***	6
MacKintosh, 2019 [57]	***		***	6
Moriconi, 2022 [59]	****	**	***	9
Moszak, 2018 [44]	***		***	6
Nikolic, 2011 [60]	***		***	6
Sarwer, 2018 [61]	***		***	6
Tussing-Humphreys, 2011 [62]	**	*	***	6
Wojciechowska-Kulik (2020) [63]	**	*	***	6

## Data Availability

The datasets utilized and analyzed for this review are available from the corresponding author upon reasonable request.

## Supplementary Materials

The following are available online at https://www.mdpi.com/article/10.3390/cancers16122197/s1, Table S1: List of terms used for literature searches; Table S2: Subgroup analysis for sources of heterogeneity based on random-effects model for association between weight loss and levels of CRP, IL-6, TNF-α, leptin, and adiponectin. Estradiol, estrone, and testosterone were assessed between all studies that reported values (no groupings).
