# Supplementary material for: Effects of Weight Loss on Key Obesity-Related Biomarkers Linked to the Risk of Endometrial Cancer: A Systematic Review and Meta-Analysis
Source: Cancers (Basel). 2024 Jun 11;16(12):2197. doi: 10.3390/cancers16122197 (PMC11201950; doi:10.3390/cancers16122197)
Supplement: Supplementary file 1 [file cancers-16-02197-s001.zip › cancers-3001630-supplementary/Tables/Supplementary Table S1.pdf]

Supplementary Table S1: List of terms used for literature searches.

Intervention Type	Searching Term
**Lifestyle**	Weight Loss [mesh] OR Weight Loss/ physiology [mesh] OR Obesity / therapy [mesh] OR Obesity, Morbid / epidemiology [mesh] OR Obesity / epidemiology [mesh] OR Body Mass Index [mesh] or Body Composition [mesh] AND Lifestyle [mesh] OR lifestyle interventions OR diet AND exercise OR diet OR exercise OR diabetes prevention program OR Calorie restriction [mesh] OR intermittent fasting OR intermittent energy restriction OR Fasting [mesh] OR behavioral interventions OR Health Behavior [mesh] AND Adipokines / metabolism [mesh] AND leptin OR adiponectin OR resistin OR sex hormones OR estrogen OR estradiol OR estrone OR testosterone OR Gonadal Steroid Hormones / metabolism [mesh] OR Inflammation / blood [mesh] OR inflammatory cytokines OR inflammation AND interleukin-6 OR interleukin-1B OR Weight Loss /immunology [mesh] OR Cytokines / blood [mesh] OR Tumor Necrosis Factor-alpha / blood [mesh] OR TNF-a OR Biomarkers / blood [mesh] OR CRP OR C-Reactive Protein / metabolism [mesh] OR hs-CRP OR VEGF OR VEGFA OR Vascular Endothelial Growth Factor A AND Ethnicity [mesh] OR Black or African American [mesh] OR White People [mesh] OR Racial Groups [mesh] OR Cultural Characteristics [mesh] AND Endometrial Neoplasms / complications [mesh] OR Endometrial Neoplasms [mesh] OR Uterine Neoplasms / pathology [mesh] OR Uterus / pathology [mesh] OR Endometrial Hyperplasia / prevention & control [mesh] AND Postmenopause [mesh] OR Middle Aged [mesh]
**Bariatric Surgery**	Weight Loss [mesh] OR Weight Loss/ physiology [mesh] OR Obesity / therapy [mesh] OR Obesity, Morbid / epidemiology [mesh] OR Obesity / epidemiology [mesh] OR Body Mass Index [mesh] or Body Composition [mesh] AND Laparoscopy [mesh] OR Obesity / surgery [mesh] OR Gastrectomy / methods [mesh] OR Gastric Bypass / methods [mesh] AND Adipokines / metabolism [mesh] AND leptin OR adiponectin OR resistin OR sex hormones OR estrogen OR estradiol OR estrone OR testosterone OR Gonadal Steroid Hormones / metabolism [mesh] OR Inflammation / blood [mesh] OR inflammatory cytokines OR inflammation AND interleukin-6 OR interleukin-1B OR Weight Loss /immunology [mesh] OR Cytokines / blood [mesh] OR Tumor Necrosis Factor-alpha / blood [mesh] OR TNF-a OR Biomarkers / blood [mesh] OR CRP OR C-Reactive Protein / metabolism [mesh] OR hs-CRP OR VEGF OR VEGFA OR Vascular Endothelial Growth Factor A AND Ethnicity [mesh] OR Black or African American [mesh] OR White People [mesh] OR Racial Groups [mesh] OR Cultural Characteristics [mesh] AND Endometrial Neoplasms / complications [mesh] OR Endometrial Neoplasms [mesh] OR Uterine Neoplasms / pathology [mesh] OR Uterus / pathology [mesh] OR Endometrial Hyperplasia / prevention & control [mesh] AND Postmenopause [mesh] OR Middle Aged [mesh]
**Pharmaceutical**	Weight Loss [mesh] OR Weight Loss/ physiology [mesh] OR Obesity / therapy [mesh] OR Obesity, Morbid / epidemiology [mesh] OR Obesity / epidemiology [mesh] OR Body Mass Index [mesh] or Body Composition [mesh] AND Glucagon-Like Peptide 1 / analogs & derivatives [mesh] OR Obesity / drug therapy [mesh] OR weight loss drugs OR Liraglutide / therapeutic use [mesh] OR Orlistat / therapy [mesh] OR Anti-Obesity Agents / therapeutic use [mesh] OR weight loss medications OR weight loss pharmacotherapies OR Gastric Inhibitory Polypeptide / therapeutic use [mesh] AND Adipokines / metabolism [mesh] AND leptin OR adiponectin OR resistin OR sex hormones OR estrogen OR estradiol OR estrone OR testosterone OR Gonadal Steroid Hormones / metabolism [mesh] OR Inflammation / blood [mesh] OR inflammatory cytokines OR inflammation AND interleukin-6 OR interleukin-1B OR Weight Loss /immunology [mesh] OR Cytokines / blood [mesh] OR Tumor Necrosis Factor-alpha / blood [mesh] OR TNF-a OR Biomarkers / blood [mesh] OR CRP OR C-Reactive Protein / metabolism [mesh] OR hs-CRP OR VEGF OR VEGFA OR Vascular Endothelial Growth Factor A AND Ethnicity [mesh] OR Black or African American [mesh] OR White People [mesh] OR Racial Groups [mesh] OR Cultural Characteristics [mesh] AND Endometrial Neoplasms / complications [mesh] OR Endometrial Neoplasms [mesh] OR Uterine Neoplasms / pathology [mesh] OR Uterus / pathology [mesh] OR Endometrial Hyperplasia / prevention & control [mesh] AND Postmenopause [mesh] OR Middle Aged [mesh]
