# Supplementary material for: Effects of Weight Loss on Key Obesity-Related Biomarkers Linked to the Risk of Endometrial Cancer: A Systematic Review and Meta-Analysis
Source: Cancers (Basel). 2024 Jun 11;16(12):2197. doi: 10.3390/cancers16122197 (PMC11201950; doi:10.3390/cancers16122197)
Supplement: Supplementary file 1 [file cancers-16-02197-s001.zip › cancers-3001630-supplementary/Tables/Supplementary Table S2.pdf]

Supplementary Table S2: Subgroup analysis for sources of heterogeneity based on random-effects model for association between weight loss and levels of CRP, IL-6, TNF-α, leptin, and adiponectin. Estradiol, estrone, and testosterone were assessed between all studies that reported values (no groupings).

Subgroup	Effect sizes (n)	Effect sizes (95% CI)	I^2 (%)	*P* heterogeneity	*P* Overall	*P* within groups	*P* between groups
**CRP Levels**							
**Overall**	**2.64**	**-3.37 (-5.88, -0.86)**	**100**	**< 0.00001**	**0.008**	**-**	**-**
**Sex Percentages**							**0.92**
100% female (Yes)	0.78	-2.80 (-9.83, 4.23)	57	0.13	-	0.44	-
100% female (No)	2.07	-3.19 (-6.22, -0.17)	100	< 0.00001	-	0.04	-
**Menopause Status**							**0.93**
Pre-Menopause	1.76	-2.96 (-6.26, 0.34)	100	< 0.00001	-	0.08	-
Post-Menopause	0.83	-3.37 (-11.29, 4.55)	100	< 0.00001	-	0.40	-
**Sample Size**							**0.17**
<500	0.66	-1.44 (-5.75, 2.86)	100	< 0.00001	-	0.51	-
>500	2.07	-6.94 (-13.49, -0.38)	100	< 0.00001	-	0.04	-
**BMI**							**0.50**
<35	10.53	-3.83 (-4.55, -3.12)	56	0.13	-	< 0.00001	-
>35	1.41	-2.58 (-6.15, -1.00)	100	< 0.00001	-	0.16	-
**Intervention Duration**							**0.14**
< 3 months	3.21	-3.30 (-5.32, -1.28)	16	0.28	-	0.001	-
3 to 5 months	5.14	-5.52 (-7.63, -3.42)	93	0.0002	-	< 0.00001	-
6 to 11 months	1.08	16.03 (-13.17, 45.23)	97	< 0.0001	-	0.28	-
≥ 12 months	2.99	-7.30 (-12.09, -2.51)	100	< 0.00001	-	0.003	-

BMI: Body Mass Index; IL-6: interleukin-6, CRP: c-reactive protein; TNF-α: tumor-necrosis factor-α

Subgroup	Effect sizes (n)	Effect sizes (95% CI)	I^2 (%)	*P* heterogeneity	*P* Overall	*P* within groups	*P* between groups
**IL-6 Levels**							
**Overall**	**1.32**	**-1.69 (-4.21, 0.83)**	**99**	**< 0.00001**	**0.19**	**-**	**-**
**Sex Percentages**							**0.77**
100% female (Yes)	0.78	-2.80 (-9.83, 4.23)	57	0.13	-	0.44	-
100% female (No)	0.90	-1.63 (-5.20, 1.94)	57	0.10	-	0.37	-
**Menopause Status**							**< 0.00001**
Pre-Menopause	7.02	-3.57 (-4.56, -2.57)	3	0.36	-	< 0.0001	-
Post-Menopause	4.95	-0.54 (-0.75, -0.32)	0	0.48	-	< 0.0001	-
**Sample Size**							-
<500	1.32	-1.69 (-4.21, 0.83)	99	< 0.00001	-	0.19	-
>500	-	-	-	-	-	-	-
**BMI**							**< 0.00001**
<35	39.66	-3.60 (-3.78, -3.42)	-	-	-	< 0.00001	-
>35	4.98	-0.54 (-0.75, -0.33)	0	0.41	-	< 0.00001	-
**Intervention Duration**							**0.26**
< 3 months	3.21	-3.30 (-5.32, -1.28)	16	0.28	-	0.001	-
3 to 5 months	-	-	-	-	-	-	-
6 to 11 months	0.50	1.30 (-3.82, 6.42)	-	-	-	0.62	-
≥ 12 months	0.78	-2.80 (-9.83, 4.23)	57	0.13	-	0.44	-

BMI: Body Mass Index; IL-6: interleukin-6, CRP: c-reactive protein; TNF-α: tumor-necrosis factor-α

Subgroup	Effect sizes (n)	Effect sizes (95% CI)	I^2 (%)	*P* heterogeneity	*P* Overall	*P* within groups	*P* between groups
**TNF-α Levels**							
**Overall**	**3.33**	**-3.62 (-5.75, -1.49)**	**85**	**< 0.00001**	**0.0009**	**-**	**-**
**Sex Percentages**							**-**
100% female (Yes)	-	-	-	-	-	-	-
100% female (No)	3.33	-3.62 (-5.75, -1.49)	85	< 0.00001	-	0.0009	-
**Menopause Status**							**0.05**
Pre-Menopause	3.82	-4.27 (-6.46, -2.08)	87	< 0.0001	-	0.0001	-
Post-Menopause	0.50	1.30 (-3.82, 6.42)	-	-	-	0.62	-
**Sample Size**							**-**
<500	3.33	-3.62 (-5.75, -1.49)	85	< 0.0001	-	0.0009	-
>500	-	-	-	-	-	-	-
**BMI**							**0.43**
<35	10.53	-3.83 (-4.55, -3.12)	56	0.13	-	< 00001	-
>35	1.27	-10.02 (-25.48, 5.45)	91	< 0.0001	-	0.20	-
**Intervention Duration**							**0.50**
< 3 months	3.21	-3.30 (-5.32, -1.28)	16	0.28	-	0.001	-
3 to 5 months	8.42	-4.40 (-5.42, -3.38)	-	-	-	< 0.00001	-
6 to 11 months	0.90	-17.87 (-56.85, 21.10)	95	< 0.00001	-	0.37	-
≥ 12 months	-	-	-	-	-	-	-

BMI: Body Mass Index; IL-6: interleukin-6, CRP: c-reactive protein; TNF-α: tumor-necrosis factor-α

Subgroup	Effect sizes (n)	Effect sizes (95% CI)	I^2 (%)	*P* heterogeneity	*P* Overall	*P* within groups	*P* between groups
**Leptin Levels**							
**Overall**	**2.40**	**-2.38 (-4.33, -0.43)**	**98**	**< 0.00001**	**0.02**	**-**	**-**
**Sex Percentages**							**0.11**
100% female (Yes)	1.73	-12.01 (-25.62, 1.60)	89	< 0.00001	-	0.08	-
100% female (No)	0.33	-0.50 (-3.46, 2.46)	98	< 0.00001	-	0.33	-
**Menopause Status**							**0.15**
Pre-Menopause	2.62	-2.72 (-4.75, -0.69)	98	< 0.00001	-	0.009	-
Post-Menopause	0.50	1.30 (-3.82, 6.42)	-	-	-	0.62	-
**Sample Size**							-
<500	2.40	-2.38 (-4.33, -0.43)	98	< 0.00001	-	0.02	-
>500	-	-	-	-	-	-	-
**BMI**							**0.43**
<35	2.14	-1.86 (-3.55, -0.16)	98	< 0.00001	-	0.03	-
>35	1.10	-6.80 (-18.88, 5.28)	96	< 0.00001	-	0.27	-
**Intervention Duration**							**< 0.00001**
< 3 months	39.66	-3.60 (-3.78, -3.42)	-	-	-	< 0.00001	-
3 to 5 months	25.58	-6.55 (-705, -6.05)	-	-	-	< 0.00001	-
6 to 11 months	0.84	-5.69 (-19.02, 7.65)	96	< 0.00001	-	0.40	-
≥ 12 months	0.48	-2.25 (-11.35, 6.85)	71	0.07	-	0.63	-

BMI: Body Mass Index; IL-6: interleukin-6, CRP: c-reactive protein; TNF-α: tumor-necrosis factor-α

Subgroup	Effect sizes (n)	Effect sizes (95% CI)	I^2 (%)	*P* heterogeneity	*P* Overall	*P* within groups	*P* between groups
**Adiponectin Levels**							
**Overall**	**3.29**	**-3.74 (-5.96, -1.51)**	**99**	**< 0.00001**	**0.0010**	**-**	**-**
**Sex Percentages**							**1.00**
100% female (Yes)	2.46	-3.25 (-5.85, -0.66)	12	0.29	-	0.01	**-**
100% female (No)	2.11	-3.24 (-6.26, -0.23)	99	< 0.00001	-	0.04	-
**Menopause Status**							**0.83**
Pre-Menopause	4.02	-3.50 (-5.21, -1.80)	97	< 0.00001	-	< 0.00001	**-**
Post-Menopause	0.83	-4.79 (-16.13, 6.56)	95	< 0.0001	-	0.41	-
**Sample Size**							**< 0.00001**
<500	3.75	-3.16 (-4.81, -1.51)	97	< 0.00001	-	0.0002	**-**
>500	35.59	-10.29 (-10.86, -9.72)	-	-	-	< 0.00001	-
**BMI**							**0.85**
<35	11.17	-3.50 (-4.12, -2.89)	84	0.002	-	< 0.00001	-
>35	1.54	-3.11 (-7.06, 0.85)	98	< 0.00001	-	0.12	-
**Intervention Duration**							**< 0.00001**
< 3 months	39.66	-3.60 (-3.78, -3.42)	-	-	-	< 0.00001	-
3 to 5 months	5.14	-5.52 (-7.63, -3.42)	93	0.0002	-	< 0.00001	-
6 to 11 months	0.08	-0.57 (-15.07, 13.93)	97	< 0.00001	-	0.94	-
≥ 12 months	35.62	-10.28 (-10.85, -9.72)	0	0.73	-	< 0.00001	-

BMI: Body Mass Index; IL-6: interleukin-6, CRP: c-reactive protein; TNF-α: tumor-necrosis factor-α

Subgroup	Effect sizes (n)	Effect sizes (95% CI)	I^2 (%)	*P* heterogeneity	*P* Overall	*P* within groups	*P* between groups
**Estradiol Levels**							
**Overall**	**1.57**	**-3.91 (-8.77, 0.96)**	**99**	**< 0.00001**	**0.12**	**-**	**-**
**Sex Percentages**							-
100% female (Yes)	1.57	-3.91 (-8.77, 0.96)	99	< 0.00001	0.12	-	-
100% female (No)	-	-	-	-	-	-	-
**Menopause Status**							-
Pre-Menopause	-	-	-	-	-	-	-
Post-Menopause	1.57	-3.91 (-8.77, 0.96)	99	< 0.00001	0.12	-	-
**Sample Size**							-
<500	1.57	-3.91 (-8.77, 0.96)	99	< 0.00001	0.12	-	-
>500	-	-	-	-	-	-	-
**BMI**							-
<35	1.57	-3.91 (-8.77, 0.96)	99	< 0.00001	0.12	-	-
>35	-	-	-	-	-	-	-
**Intervention Duration**							-
< 3 months	-	-	-	-	-	-	-
3 to 5 months	-	-	-	-	-	-	-
6 to 11 months	-	-	-	-	-	-	-
≥ 12 months	1.57	-3.91 (-8.77, 0.96)	99	< 0.00001	0.12	-	-

BMI: Body Mass Index; IL-6: interleukin-6, CRP: c-reactive protein; TNF-α: tumor-necrosis factor-α

Subgroup	Effect sizes (n)	Effect sizes (95% CI)	I^2 (%)	*P* heterogeneity	*P* Overall	*P* within groups	*P* between groups
**Estrone Levels**							
**Overall**	**8.07**	**-2.88 (-3.58, -2.18)**	**0**	**0.58**	**< 0.00001**	**-**	**-**
**Sex Percentages**							**-**
100% female (Yes)	8.07	-2.88 (-3.58, -2.18)	0	0.58	< 0.00001	-	-
100% female (No)	-	-	-	-	-	-	-
**Menopause Status**							**-**
Pre-Menopause	-	-	-	-	-	-	-
Post-Menopause	8.07	-2.88 (-3.58, -2.18)	0	0.58	< 0.00001	-	-
**Sample Size**							**-**
<500	8.07	-2.88 (-3.58, -2.18)	0	0.58	< 0.00001	-	-
>500	-	-	-	-	-	-	-
**BMI**							**-**
<35	8.07	-2.88 (-3.58, -2.18)	0	0.58	< 0.00001	-	-
>35	-	-	-	-	-	-	-
**Intervention Duration**							-
< 3 months	-	-	-	-	-	-	-
3 to 5 months	-	-	-	-	-	-	-
6 to 11 months	-	-	-	-	-	-	-
≥ 12 months	8.07	-2.88 (-3.58, -2.18)	0	0.58	< 0.00001	-	-

BMI: Body Mass Index; IL-6: interleukin-6, CRP: c-reactive protein; TNF-α: tumor-necrosis factor-α

Subgroup	Effect sizes (n)	Effect sizes (95% CI)	I^2 (%)	*P* heterogeneity	*P* Overall	*P* within groups	*P* between groups
**Testosterone Levels**							
**Overall**	**0.89**	**-1.12 (-3.60, 1.36)**	**89**	**.003**	**0..8**	**-**	**-**
**Sex Percentages**							-
100% female (Yes)	0.89	-1.12 (-3.60, 1.36)	89	.003	0..8	-	-
100% female (No)	-	-	-	-	-	-	-
**Menopause Status**							-
Pre-Menopause	-	-	-	-	-	-	-
Post-Menopause	0.89	-1.12 (-3.60, 1.36)	89	.003	0..8	-	-
**Sample Size**							-
<500	0.89	-1.12 (-3.60, 1.36)	89	.003	0..8	-	-
>500	-	-	-	-	-	-	-
**BMI**							-
<35	0.89	-1.12 (-3.60, 1.36)	89	.003	0..8	-	-
>35	-	-	-	-	-	-	-
**Intervention Duration**							-
< 3 months	-	-	-	-	-	-	-
3 to 5 months	-	-	-	-	-	-	-
6 to 11 months	-	-	-	-	-	-	-
≥ 12 months	0.89	-1.12 (-3.60, 1.36)	89	.003	0..8	-	-

BMI: Body Mass Index; IL-6: interleukin-6, CRP: c-reactive protein; TNF-α: tumor-necrosis factor-α
